# Sequential Immunizations with heterosubtypic virus-like particles elicit cross protection against divergent influenza A viruses in mice

**DOI:** 10.1038/s41598-018-22874-w

**Published:** 2018-03-15

**Authors:** Yuan Luo, Teena Mohan, Wandi Zhu, Chao Wang, Lei Deng, Bao-Zhong Wang

**Affiliations:** Center for Inflammation, Immunity & Infection, Institute for Biomedical Sciences, Georgia State University, 100 Piedmont Avenue SE, Atlanta, GA 30303-5090 USA

## Abstract

Seasonal influenza vaccines have proven to be effective against well-matched viruses in healthy adults. However, rapid accumulation of mutations in the main antigenic surface proteins of influenza can compromise the efficiency of flu vaccines. Occasionally, influenza pandemics arise and present a different type of challenge to current seasonal vaccines. Novel vaccination strategies that can educate the host immune system to generate immune responses focusing on conserved epitopes on theses antigenic surface proteins are crucial for controlling and limiting influenza epidemics and pandemics. In this study, we have sequentially vaccinated mice with heterosubtypic influenza HA virus-like particles (VLPs) harboring H1, H8, and H13 from the HA phylogenetic group 1, or H3, H4, and H10 from the HA phylogenetic group 2, or in various combinations. The immunized animals were fully protected when challenged with lethal doses of heterosubtypic viruses from either phylogenetic group. Our vaccination approach demonstrates a promising strategy for the development of a ‘universal influenza vaccine’.

## Introduction

Seasonal influenza epidemics and occasional pandemics remain a public health concern throughout the world. Under non-pandemic conditions, about 200,000–500,000 deaths are associated with influenza infection each year. Influenza causes over 40,000 deaths every year in the United States alone^1^. In a pandemic, such as the case of 1918, the global mortality could be in millions^2^. Influenza has been and continues to be a severe threat to public health.

The most effective way to protect against influenza is through vaccination. However, the current vaccination approaches rely on achieving a good match between circulating viruses and the vaccine strains^3^. Mismatches may occur because the circulating viruses rapidly accumulate mutations in genes encoding the major surface antigens hemagglutinin (HA) and neuraminidase (NA)^4^. Challenges may also include the reassortment of viral gene segments between different viruses of human or zoonotic origin, which may lead to the emergence of totally new and highly pathogenic strains^5^. An ideal ‘universal vaccine’ could resolve all these problems by eliciting broadly reactive immune responses targeting conserved epitopes shared by all influenza viruses^6,7^.

Although tremendous efforts are under way, no universal influenza vaccine has been fully developed. While broadly neutralizing antibodies (bnAbs) are ideal for protective immunity and the generation of such antibodies could result in universal protection, they are rare and difficult to produce via vaccination. Vaccinations with the seasonal vaccine formulations typically skew the specificity of B cell responses towards strain-specific epitopes. The generation of bnAb immune responses relies on guiding the human immune system to recognize conserved but not strain-specific epitopes. The induction of bnAbs via vaccination thus must educate the host immune system to ignore the highly immunogenic strain-specific epitopes but to focus on the less immunogenic conserved ones^8,9^.

Recently, several bnAbs were isolated from cases of natural infection and vaccination in humans and mice^9,10^. Successive infections with live viruses lead to a reduction in strain-specific antibody titers against the most recent strain while nurturing broader epitope-specific antibody titers^4,11–13^. These studies into optimal immunogen designs and iterative antigenic exposure provide important novel insights into the development of immune responses for creating a successful ‘universal influenza vaccine’. These observations strongly suggest that sequential infections or vaccinations play a central role in the induction of broadly cross-reactive antibodies. In the present report, we have designed a cross-subtypic sequential vaccination strategy with different subtypic influenza virus-like particles (VLPs) containing HA from H1N1, H8N4, H13N6 (HA phylogenetic group 1) or H3N2, H4N6, and H10N2 (HA phylogenetic group 2) viral strains or in combination, to elicit broad protection against divergent viruses in the same HA phylogenetic group or both groups.

## Results

### Characterization of influenza VLPs

**Figure 1:**
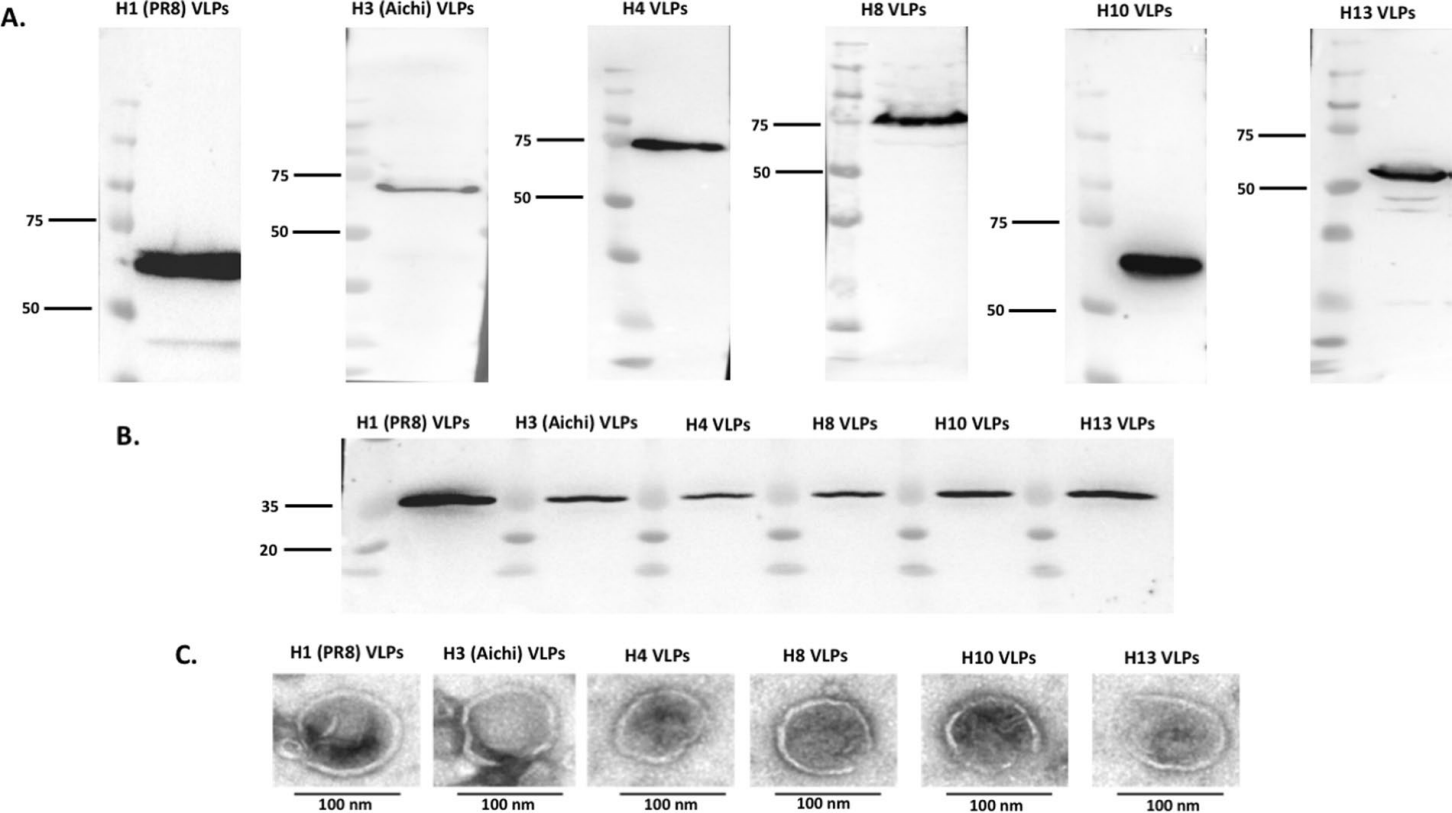
Characterization of purified VLPs. Figure represents Western blot of (**A**) HA and (**B**) M1 proteins in the different VLPs. Total HA and M1 contents in the prepared VLPs were analyzed by SDS-PAGE followed by Western blot using mouse anti-influenza HA and anti-M1 antibodies, respectively, (**C**) Negative staining electron micrographs of different influenza HA VLPs. Bars represent ∼100 nm.

HA/M1 VLPs were purified by sucrose density gradient centrifugation as described previously^14,15^. Western blot was performed to analyze the expression of HA and M1 proteins in the prepared VLPs using their specific antibodies. Characteristic bands with molecular weights of 55–70 KD (Fig. 1A) and ∼25 KD (Fig. 1B) were observed for HA and M1 proteins, respectively, which were consistent with the molecular weights estimated from their amino acid compositions. TEM data showed that the prepared VLPs were spherical in morphology with a diameter of ∼100 nm (Fig. 1C), which was similar with previous studies^16,17^. These results suggest that influenza HA VLPs were successfully produced for immunizations.

### Sequential immunizations with different subtypic VLPs induce broader antibody responses

Previous influenza^18–20^ and HIV^21,22^ studies have shown that sequential immunizations with modified antigens elicited bnAb responses that conferred complete or partial protection. A recent study reported that a VLP vaccine, composed of a cocktail of different antigens, protects mice from multiple subtypes of Influenza A virus^23^. An antibody response may result in changes in the distribution of IgG subclasses and measuring IgG subclasses levels is essential in understanding the B cell somatic hypermutation and subclass-switching^16^. To determine whether sequential immunizations with influenza VLPs induce cross-protection, the groups of mice were sequentially immunized with different subtypic influenza VLPs or a mixture of various VLPs through an intranasal (*i.n*.) route every 3^rd^ week. Antigen-specific IgG and IgG subtypes in immune sera and IgA in mucosal samples were measured at every 2^nd^ week post-vaccination in mice immunized with different vaccine formulations.

**Figure 2:**
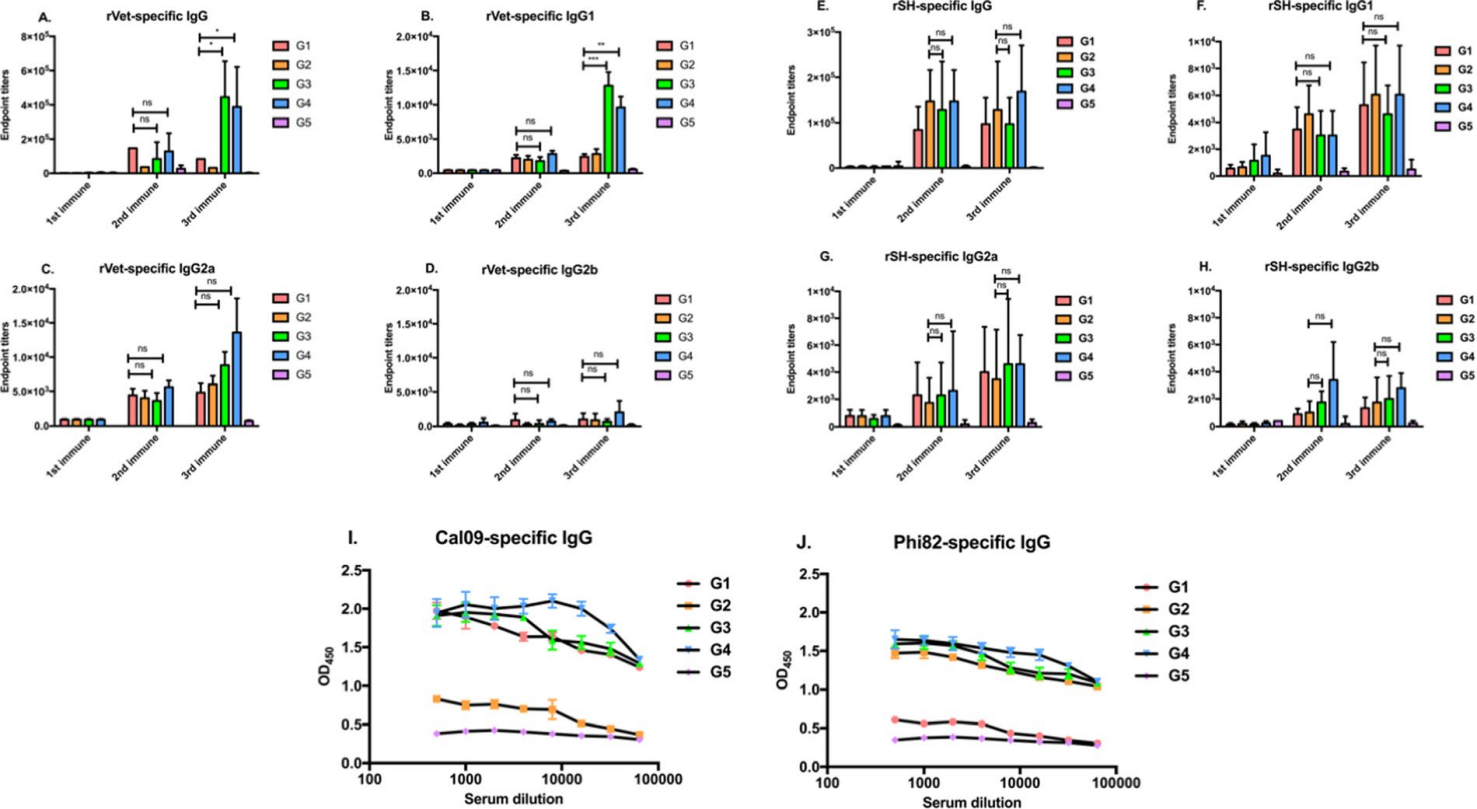
rVet and rSH- specific serum IgG/IgG subtype endpoint titers and intracellular ELISA. Endpoint titers are expressed as the highest dilution of serum having a mean OD_450_ greater than the mean plus 2 standard deviation of similarly diluted naïve serum samples. For intracellular ELISA, MDCK cells were exposed to Cal09 or Phi82 virions, used as antigens in the ELISA. After permeabilization, MDCK cells were incubated with diluted sera, followed by detection with HRP-conjugated anti-mouse IgG antibody. Figure represents (**i**) rVet-specific (**A**) IgG, (**B**) IgG1, (**C**) IgG2a, and (**D**) IgG2b; (**ii**) rSH-specific (**E**) IgG, (**F**) IgG1, (**G**) IgG2a, and (**H**) IgG2b; and (**iii**) (**I**) Cal09-specific and (**J**) Phi82-specific IgG levels in sera. Results are represented as the mean ± SD (n = 5). (*P < 0.05, **P < 0.01, ***P < 0.001, ns-non-significant).

After the 2^nd^ immunization, reassortant A/Vietnam/1203/2004 (rVet) (H5N1)-specific total IgG levels were comparable in all the groups and did not show any increase in their total IgG levels. We observed that after the 3^rd^ vaccination, the total virus-specific IgG endpoint titers were significantly (P < 0.05) increased in groups 3 and 4. Meanwhile, no significant differences were found in the other vaccination groups (Fig. 2A). These results suggest that the immunizations of animals of group 3 and 4 enhanced the rVet-specific serum antibody levels in the systemic circulations. To further demonstrate the humoral immune responses, we measured endpoint titers of serum IgG subtypes, *i.e*. IgG1, IgG2a and IgG2b, after each vaccination. Group 1, 2, and 5 did not show any increase in either IgG1 or IgG2a/2b antibody levels. IgG1 and IgG2a levels in the animals of group 3 and 4 were significantly (P < 0.001) induced when compared to group 1. On the other side, groups 3 and 4 did not support the induction of IgG2b antibody levels. These data demonstrated that groups 3 and 4 showed mixed Th1 and Th2 type of immune responses (Fig. 2B–D). Furthermore, we also evaluated reassortant A/Shanghai/02/2013 (rSH) (H7N9)-specific total serum IgG and IgG subtype levels in mice vaccinated with various vaccine formulations. IgG endpoint titers were found increased after the 2^nd^ vaccination when compared to rSH IgG results. Although the level of IgG and IgG subtypes were increased after immunization, no significant differences were observed between the 2^nd^ and 3^rd^ immunization or when compared with group 2 (P > 0.05) (Fig. 2E–H). For IgG subtypes, all the VLP immunized animals showed no change in both the rSH-specific IgG1 and IgG2a/2b endpoint titers. To determine if sequential immunizations can elicit antibodies against A/California/07/2009 (Cal09) (H1N1) or A/Philippines/02/1982 (Phi82) (H3N2), intracellular ELISA was performed as described in the material and methods. Results showed that Cal09- (Fig. 2I) and Phi82-specific antibodies were enhanced more specifically in the sequentially immunized animal groups (Fig. 2J). Taken together, these results suggested that sequential immunizations can induce heterosubtypic serum IgG levels.

### Sequential immunizations with different influenza VLPs enhance mucosal immune responses

**Figure 3:**
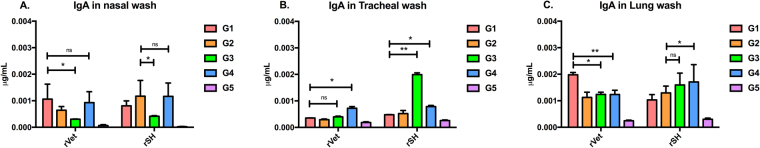
Mucosal responses in nasal, tracheal, and lung washes. After the 3^rd^ immunization, (**A**) nasal wash, (**B**) tracheal wash, and (**C**) lung wash were collected and total antigen-specific IgA amount were determined by ELISA using rVet and rSH viruses as coating antigens. Results represent the mean ± SD (n = 5). (*P < 0.05, **P < 0.01, ns-non-significant).

Because influenza viruses infect hosts through the respiratory tract, it is essential to evaluate mucosal immune responses. Two-weeks after the final immunization, nasal, tracheal, and lung washes were collected from the vaccinated animals and rVet and rSH-specific IgA levels were measured in nasal, tracheal, and lung secretions. The levels of nasal IgA against rVet in group 1 were found to be significantly (P < 0.05) higher than that of group 3, and comparable with that of group 4. rSH-specific nasal IgA levels in group 2 were significantly (P < 0.05) higher than that of group 3, and comparable with that of group 4 (Fig. 3A). We observed that the highest levels of rVet-specific IgA were induced in group 4 when compared to other vaccination groups in tracheal washes. On the other side, the highest rSH-specific tracheal IgA levels were detected in group 3, which were significantly (P < 0.01) higher than the levels observed in other groups (Fig. 3B). In lung secretions, rVet-specific IgA levels were enhanced significantly in mice of group 1 when compared with animals of groups 3 (P < 0.05) and 4 (P < 0.01). Lung IgA levels against rVet in group 4 were observed significantly (P < 0.05) higher than group 2 (Fig. 3C).

### Sequential immunizations with different VLPs increases HAI activity

**Figure 4:**
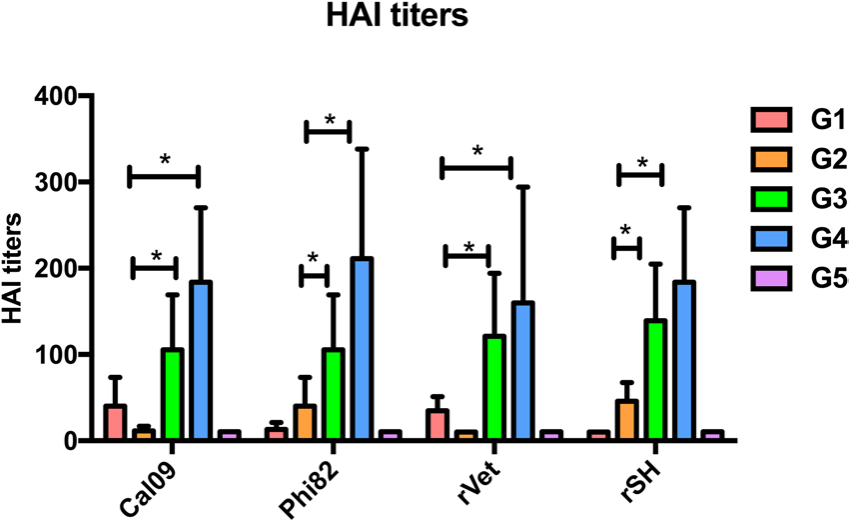
Serum HAI titers. Mean HAI titers of serum IgG was measured as described in materials and methods using MDCK cells. Results are represented as the mean ± SD (n = 5). (*P < 0.05).

HAI titers are correlated to the protective efficacy of influenza antibody responses. To further compare the induced antibody responses, we determined the serum HAI titers against Cal09, Phi82, rVet, and rSH viruses. As shown in Fig. 4, both group 3 and group 4 have significantly (P < 0.05) higher HAI titers compared to group 1, *i.e*. Cal09 (H1N1) and rVet (H5N1), or group 2, *i.e*. Phi82 (H3N2) and rSH (H7N9).

### Sequential immunization promotes virus-specific T cell responses

**Figure 5:**
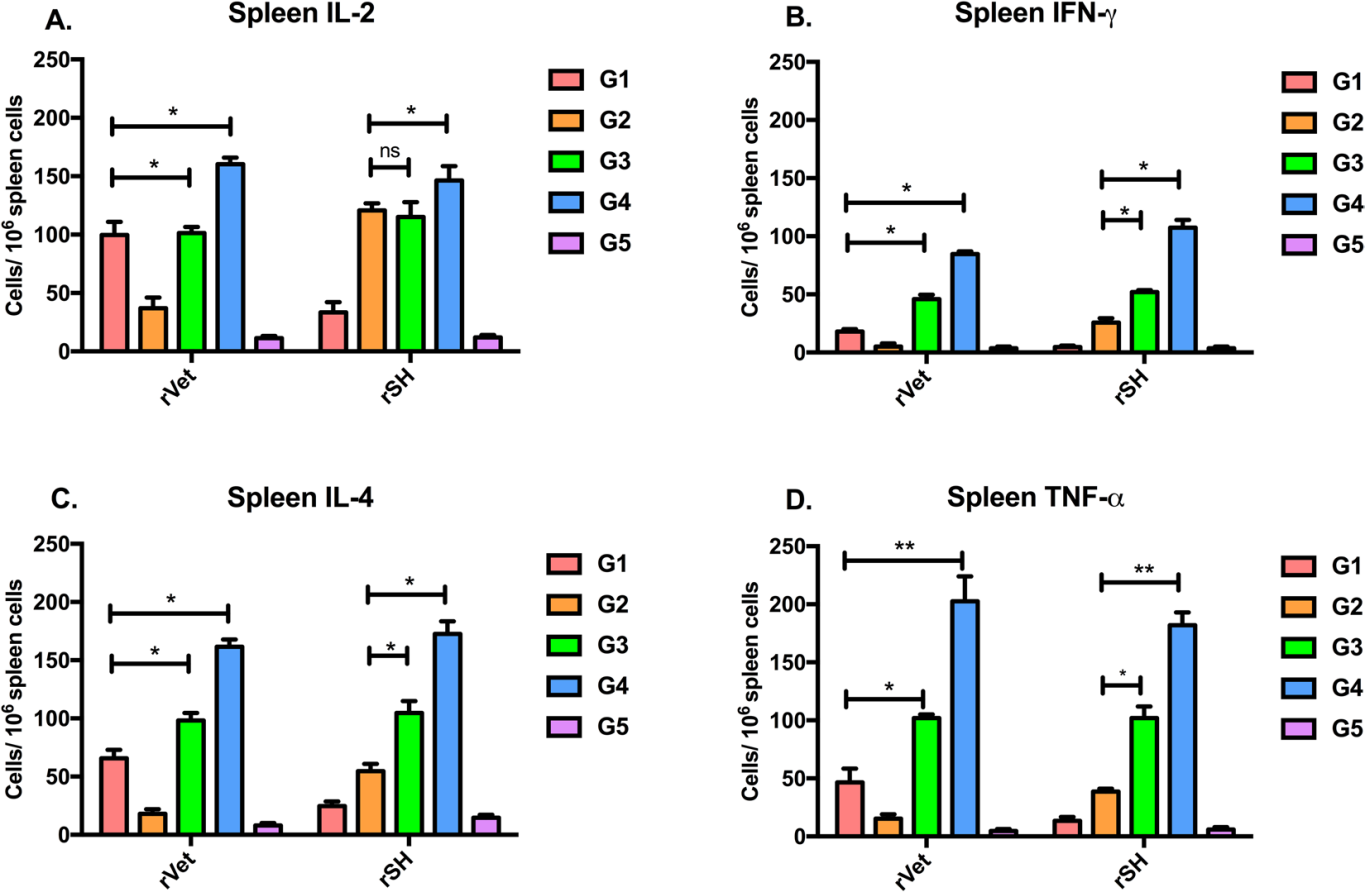
Estimation of cytokine levels. Splenocytes were isolated from immunized mice 3 week after the final immunization. Cells (1 × 10^6^) were seeded into 96-well culture plates. The inactivated rVet or rSH viruses were added into each well and secreted (**A**) IL-2, (**B**) IFN-γ, (**C**) IL-4, and (**D**) TNF-α levels were determined as described in materials and methods. Results are represented as the mean ± SD (n = 5). (*P < 0.05, **P < 0.01).

Next, we determined the H5N1 and H7N9 virus-specific induced cytokine levels using ELISPOT. In rVet and rSH-specific IL-2 secretions, a significant (P < 0.05) increase in IL-2 levels in groups 3 and 4 were found when compared with group 1 and 2, respectively (Fig. 5A). Mice of groups 3 and 4 showed significantly (P < 0.05) enhanced levels of IFN-γ while the IFN-γ levels were undetectable in group 1 and 2 (Fig. 5B). We also observed that IL-4 levels were significantly (P < 0.05) increased in both groups 3 and 4 compared to the animals vaccinated with group 1 or 2 in rVet and rSH-specific responses, respectively (Fig. 5C). Like other cytokine levels, rVet and rSH-specific TNF-α levels was also increased significantly (P < 0.05) in both groups 3 and 4 compared to group 1 and 2, respectively (Fig. 5D). PBS immunized mice were not able to induce cytokine-producing lymphocytes specific to either of the viruses. These results suggest that sequential immunization induces both Th1 and Th2 types of cytokine levels.

### Sequential immunization reduces post-challenge inflammatory cytokine levels

**Figure 6:**
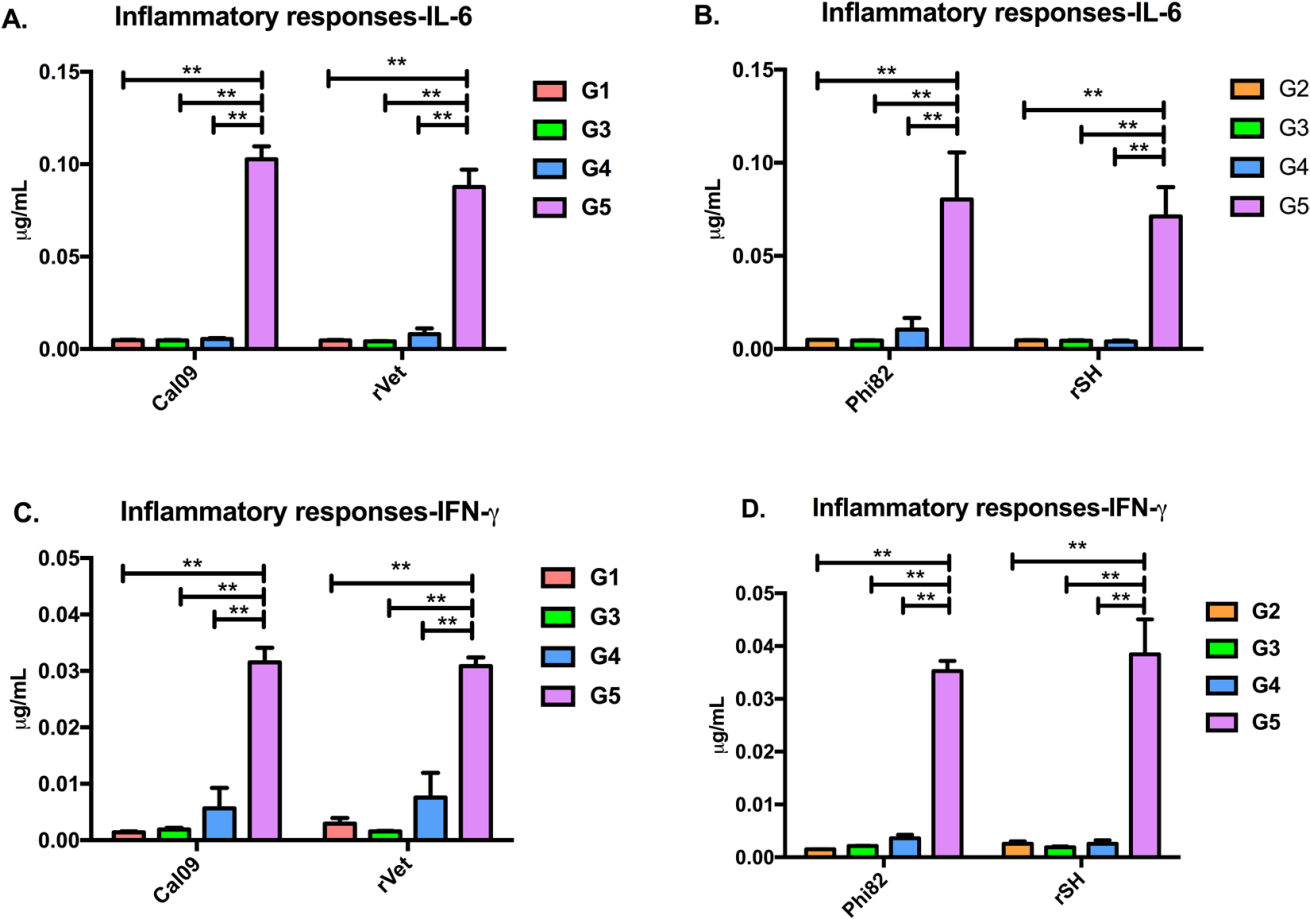
Post inflammatory response. The levels of inflammatory cytokines; (**A**,**B**) IL-6, and (**C**,**D**) IFN-γ were evaluated in the lung samples after the virus challenges. Lung samples were prepared on day 4 post-challenge and the cytokines were estimated by ELISA. Results are represented as the mean ± SD (n = 5). (*P < 0.05, **P < 0.01).

Tissue damage is always relative to the levels of inflammatory cytokines^24^. The elevated levels of proinflammatory cytokines (TNFα, IL-1β and IL-10) during influenza infection have also been primarily associated with increased lung pathology and worse outcomes, but it is uncertain whether some of these cytokines could also be protective during seasonal influenza infection^25^. On the other hand, IL-6 and IFN-γ specifically support protection against viral infections^26,27^. At day 4 post-challenge, IL-6 and IFN-γ levels were measured by sandwich ELISA in lung tissue. IL-6 and IFN-γ levels were significantly higher in PBS immunized mice after being infected with Cal09, Phi82, rVet or rSH. On the contrary, very low levels of IL-6 and IFN-γ were detected in animals of both groups 3 or 4 than other groups of animals (Fig. 6A–D). These data show that groups 3 and 4 had low levels of inflammatory cytokines in the lungs after different virus infections.

### Sequential immunization contributes to broad protection with less body weight changes against heterologous and heterosubtypic viruses

**Figure 7:**
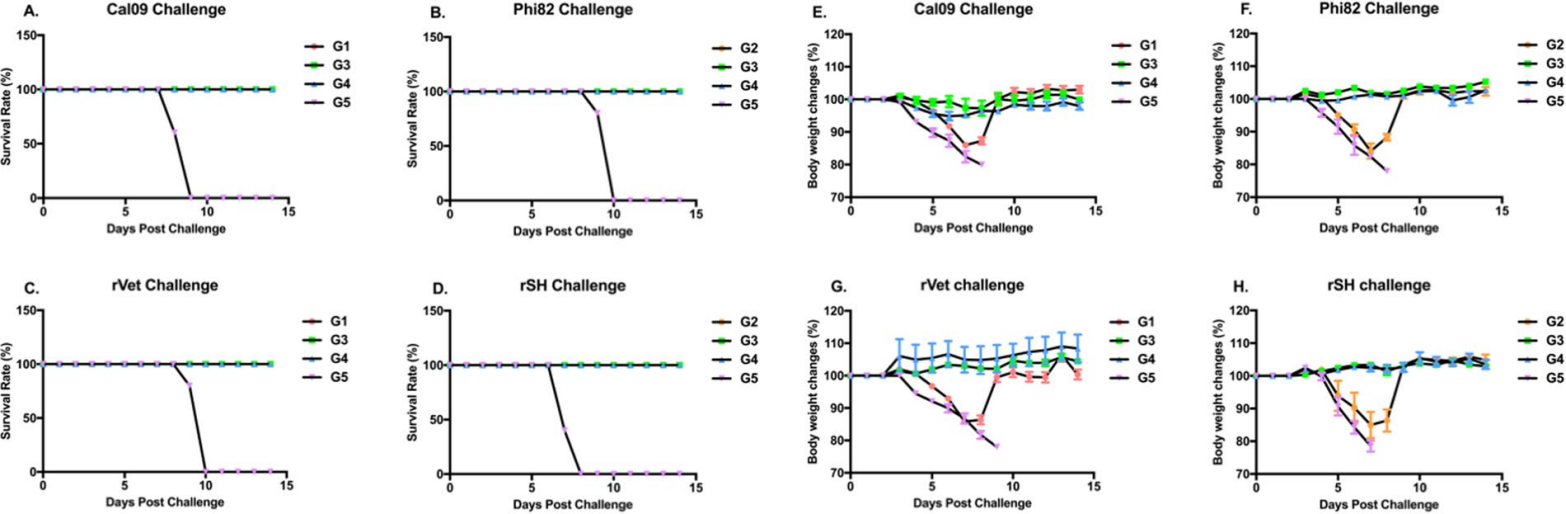
Challenges study. Vaccinated animals were challenged with 10x LD50 mouse-adapted viruses. Mice were monitored daily for 14 days for body weight changes and their survival. Figures show (**i**) the survival of vaccinated animals challenged with (**A**) Cal09 (H1N1), (**B**) Phi82 (H3N2), (**C**) rVet (H5N1), or (**D**) rSH (H7N9) viruses; and (**ii**) body weight changes in the vaccinated animals challenged with (**E**) Cal09, (**F**) Phi82, (**G**) rVet, or (**H**) rSH viruses.

To determine whether sequential immunizations protect mice from lethal virus challenge, vaccinated animals were challenged with 10 × LD_50_ of mouse adapted Cal09, Phi82, rVet, or rSH viruses. Interestingly, mice sequentially vaccinated with influenza VLPs in different formulations showed complete (100%) protection towards all the viruses used for challenges (Fig. 7A–D). During body weight analysis, the body weight of mice of group 3 began to drop at day 4 post-challenge, similar with what happened in the PBS immunized animals. But at day 7, the sequentially immunized group of animals showed recovery and began to regain body weight (Fig. 7D–G). These results showed that the animals of groups 3 and 4 developed complete protection from heterologous and heterosubtypic virus challenges and maintained their body weight throughout the study when compared to the negative control groups.

## Discussion

Immunity to influenza viruses is currently achieved by vaccination with strains representing those predicted to circulate in the coming flu season. In a healthy person, the virus acts as a robust immunogen, eliciting antibody titers that correlate with *in vivo* protection. However, wild influenza viruses are continuously undergoing change and the predominant strains are constantly replaced by variants that have undergone sufficient antigenic changes to evade existing immunity.

Many research teams have sought to create a ‘universal vaccine’ by inducing responses to shared conserved epitopes, including bnAbs, that could provide long-lasting protective immunity to multiple viral strains. However, the generation of broadly reactive responses towards the conserved epitopes on HA is the main obstacle to the development of a ‘universal vaccine’. The idea behind sequential vaccination is to divert the host’s immune response from dominant-but-variable epitopes to more conserved antigenic sites. In the current report, we have evaluated sequential *i.n*. immunizations of mice with different subtypic influenza VLPs and observed a high level of elicited heterologous and heterosubtypic antibody responses that subsequently provided complete protection against lethal viral challenges.

Following infection or vaccination, the induction of protective immunity against invading pathogens depends on the generation of an appropriate type of immune response through affinity maturation which is the key in generating bnAbs. Affinity maturation takes place in germinal centers (GC) where antigens are transferred by antigen-presenting cells (APCs)^28^. During antigen presentation to B cells, somatic hypermutation occurs and B cells with higher affinity are screened and later differentiate into memory B cells^29^. When stimulated with similar antigen, these memory B cells quickly amplify and secret bnAbs to clear the specific antigen. This strategy is difficult to apply when dealing with highly variable antigens, such as those found on influenza virus or HIV. Influenza’s HA stalk domain is very conserved but heavily masked by the highly variable head domain^30–32^. This conserved stalk is the main target for the generation of bnAbs^33–36^.

Recently, influenza specific bnAbs have been isolated from immunized mice, phage-display libraries, memory B cells, and plasma cells^33,35,37–44^. While almost all antibodies elicited to a traditional vaccine are strain-specific and head-domain-specific, most of the bnAbs discovered react with the stalk domain. Researchers have begun to focus their efforts on designing an antigen that can elicit stalk-specific bnAbs because the stalk domain is highly conserved between HA antigens of different subtypes^36,45^. Many studies have shown that the stalk domain can be expressed without the head region and in its natural conformation after minimal mutations to the gene^34,46–48^. This truncated protein elicits antibodies with relatively low efficacy due to the low immunogenicity of the HA stalk domain^46,47,49–51^. Part of the search for any type of universal vaccine revolves around identifying these conserved epitopes and boosting the natural immune response to them.

In the current report, we have sequentially immunized mice with native influenza HA VLPs mimicking the natural structure of the stalk domain and then challenged with live viruses. Our results demonstrate that sequentially immunized animals generated broader antibody responses and showed complete protection against various homosubtypic and heterosubtypic influenza viruses. Our results demonstrate that using multiple antigen variants with high genetic diversity can elicit bnAbs, demonstrated by the increased serum neutralizing activity. Similar studies of influenza virus has also found that immune or infection history profoundly affects broadly protective B cell immune responses^12,52^. In conclusion, sequential immunizatons with diverse HA VLPs educate the host immune system to generate broad reactive immunity conferring immune protection against challenges by different viruses in mice.

## Methods

### Cell lines and viruses

Spodoptera frugiperda (SF9) insect cells (ATCC: CRL-1711), and Madin Darby Canine Kidney (MDCK) cells (ATCC: PTA-6500) were maintained as described previously^17^. For challenge studies, mouse adapted A/California/07/2009 (Cal09) (H1N1) and A/Philippines/02/1982 (Phi82) (H3N2) viruses were prepared as described previously^53^. The rVet and rSH viruses were kindly gifted from Dr. Sang-Moo Kang, Georgia State University, Atlanta, USA. The reassortant viruses contain HA and NA genes from A/Vietnam/1203/2004 (H5N1) and A/Shanghai/02/2013 (H7N9) viruses but remaining backbone genes from A/Puerto Rico/08/1934 (H1N1). The LD_50_ (Lethal dose inducing 50% mortality) and TCID_50_ (Tissue culture infectious dose infecting 50% cells) of these strains were determined by infection of mice with serial viral dilutions and calculated by the Reed and Muench method.

### VLPs production

The recombinant baculoviruses (rBVs) expressing M1 and full-length HA genes from A/PR/08/1934 (H1N1), A/Aichi/02/1968 (H3N2), A/mallard/Netherlands/01/1999 (H4N6), A/mallard/Sweden/24/2002 (H8N4), A/mallard/Sweden/51/2002 (H10N2), and A/black-headed gull/Sweden/1/1999 (H13N6) (BEI resources, Manassas, VA, USA), were generated using the Bac-to-Bac insect cell protein expression system (Invitrogen, Carlsbad, CA, USA). Influenza VLPs (HA/M1 VLPs) were produced by co-infection of SF9 cells with rBVs expressing HA and M1 as described previously at a multiplicity of infection (MOI) of 4 and 2, respectively^54^. At 60 h post-infection, VLPs were concentrated from the cell culture supernatants by porous fiber filtration using an ÄKTA Flux (GE Healthcare, Uppsala, Sweden) and further purified by sucrose density gradient centrifugation as described previously^55^. The VLP protein concentration was determined by MicroBCA protein assay (Thermo Fisher Scientific, Waltham, MA, USA). The HA, and M1 protein profiles in VLPs were analyzed by Western blot using HA-specific (BEI resources, Manassas, VA, USA) or M1-specific (Serotec, Kidlington, UK) antibodies. Total endotoxin or bio-containment levels were analyzed by Limulus Amebocyte Lysate (LAL) test. A quantitative ELISA was done as described previously to determine HA contents in VLPs, using recombinant HA (Sino Biological Inc., North Wales, PA, USA) as the calibration standards^23^. The quality and purity of VLPs were confirmed by transmission electron microscopy^51^ (Zeiss, Oberkochen, Germany) studies^56^.

### Ethics statement

This study was carried out in strict accordance with the recommendations in the Guide of the Care and Use of Laboratory Animals of the National Institutes of Health (NIH). All animal studies were approved by the Institutional Animal Care and Use Committee (IACUC) of Georgia State University. The 6–8-week old, healthy Balb/c female mice were purchased from the Jackson Laboratory and housed in the animal facility. Immunizations and sample collections were performed under mild anesthesia that was induced and maintained with ketamine hydrochloride and xylazine. This study was performed in a BSL-2 laboratory. Mouse immunization and challenge studies were carried out in a ABSL-2 laboratory. Studies related to the use of reassortant influenza viruses (rSH and rVet) were done in BSL-2 plus and ABSL-2 plus biosafety containment levels. All these studies were approved by the Institutional Biosafety Committee of Georgia State University under protocol number B1617.

### Immunization and challenges of animals

Groups of 5–6 animals were immunized sequentially through an *i.n*. route with various influenza VLP formulations containing 10 μg of HA in total from different subtypes per immunization as shown in the table. Briefly, we sequentially immunized mice with different phylogenetic group 1 HA VLPs (group 1); phylogenetic group 2 HA VLPs (group 2); with a mixture of two HA VLPs from phylogenetic group 1 and group 2 (group 3); with a mixture of all of six types of both phylogenetic group 1 and group 2 HA VLPs (group 4); or with PBS as negative control (group 5) (Table 1). The HA antigens chosen from phylogenetic group 1 and group 2 were selected to maximize genetic divergence. This type of antigenic selection may increase the possibility to get more specific and effective antibody responses mainly against highly pathogenic strains such as H5N1 or H7N9. Mice were immunized at weeks 0, 3 and 6. One month after the last vaccination, animal groups were challenged intranasally with 10 × LD_50_ of live mouse adapted A/California/07/2009 (Cal09, H1N1), A/Philippines/2/1982 (Phi82, H3N2), rVet (H5N1), or rSH (H7N9) viruses (Table 1). For rVet, the HA and NA genes were derived from A/Vietnam/1203/2004 (H5N1, Vet) and the remaining backbone genes from A/Puerto Rico/8/1934 (H1N1, PR8) and for rSH, the HA and NA genes were derived from A/Shanghai/2/2013 (H7N9, SH), and the remaining backbone genes from A/Puerto Rico/8/1934 (H1N1, PR8)^57^. Vaccination and control groups were monitored up to 14 days for body weight changes and mortality. Mice that lost more than 20% of their original body weight were terminated and recorded as dead^55^. For post-challenge inflammatory responses, the levels of various inflammatory cytokines were measured at day 4 post challenge using ELISA in BALF without any prior *in vitro* stimulation.

**Table 1:** Schematic representation of sequential vaccination and challenges.

Groups	First Immunization	Second Immunization	Third Immunization	Challenge viruses
Group 1	H1 VLP	H8 VLP	H13 VLP	H1N1(Cal09) H5N1 (rVet)
Group 2	H3 VLP	H4 VLP	H10 VLP	H3N2(Phi82) H7N9 (rSH)
Group 3	H1 VLP + H3 VLP	H8 VLP + H4 VLP	H13 VLP + H10 VLP	H1N1(Cal09) H5N1 (rVet) H3N2 (Phi82) H7N9 (rSH)
Group 4	Mixture	Mixture	Mixture	H1N1(Cal09) H5N1 (rVet) H3N2 (Phi82) H7N9 (rSH)
Group 5	PBS	PBS	PBS	H1N1(Cal09) H5N1 (rVet) H3N2 (Phi82) H7N9 (rSH)

### Sample collection

At every 2^nd^ week post-vaccination, blood samples were collected via the submandibular veins of anesthetized animals and allowed to clot for 10 min at 37 °C. Sera and mucosal samples, including nasal, tracheal, and lung washes, were collected as described previously^54^. For ELISPOT assays, splenocytes were also collected from the sacrificed mice. For measuring post-challenge inflammatory responses, animals were sacrificed for lung tissues prior to challenge or at day 4 post-challenge as described previously^55^.

### Evaluation of humoral immune responses

The rVet and rSH virus-specific IgG, IgG subtypes (IgG1, IgG2a, and IgG2b), and IgA were determined by ELISA in sera and mucosal washes, respectively, as described previously^17^. To detect H1N1 and H3N2 virus-specific antibody levels, intracellular ELISA was performed as described in a previous study^58^. Briefly, MDCK cells were grown on 96-well flat bottom microtiter plates (Nunc-Immuno Plate Maxisorp; Nunc Life Technologies, Basel, Switzerland), and exposed to Cal09 or Phi82 virions at MOI of 5 PFU/cell. After removing the unbound viruses, cells were fixed with 4% formaldehyde. After permeabilizing and blocking the cells, serially diluted sera were added to each well. For ELISA or intracellular ELISA, color was developed using horseradish peroxidase (HRP)-labeled goat anti-mouse IgG, IgG1, IgG2a, IgG2b, and IgA antibodies (Southern Biotech, Birmingham, AL, USA). The optical density at 450 nm (OD_450_) was read with an ELISA reader (BioTek, Winooski, VT, USA). The highest dilution which gave an OD_450_ two-fold higher than that of the naïve group without dilution was determined as the antibody endpoint titer.

### Hemagglutinin inhibition assay

The hemagglutinin inhibition (HAI) titers against Cal09, Phi82, rVet, or rSH viruses were determined as recommended by the World Health Organization (WHO) and the Center for Disease Control and Prevention (CDC)^59^.

### Cytokine assay

Using quantitative ELISA, the levels of Th1/Th2 cytokines including IFN-γ, IL-2, IL-4, and TNF-α were analyzed from the culture supernatants of splenocytes, collected at 48 and 72 h of incubation. Freshly isolated splenocytes (1 × 10^6^ cells) were added to each well and stimulated with inactivated rVet and rSH at a concentration of 4 μg/ml as described earlier^17,60^.

### Statistical analysis

The data for virus-specific antibody levels, HAI titers, and inflammatory cytokines were analyzed by paired student t-tests (n = 5). All the experiments *with five mice in each group (n* = *5)* were done three times independently to check the reproducibility and the results were expressed as mean ± standard deviation (SD). Survival differences were evaluated by Log Rank Mantel-Cox test. Levels of significance (P value) were compared between PBS, and the other vaccination groups. Tests were performed using GraphPad Prism 5 software (San Diego, CA, USA). P values of <0.05 (P < 0.05) were statistically significant.
